# Assessment of physical inactivity and locomotor dysfunction in adults with visual impairment

**DOI:** 10.1038/s41598-018-30599-z

**Published:** 2018-08-13

**Authors:** Sachiko Inoue, Motoko Kawashima, Yoshimune Hiratsuka, Tadashi Nakano, Hiroshi Tamura, Koichi Ono, Akira Murakami, Kazuo Tsubota, Masakazu Yamada

**Affiliations:** 1 Department of Ophthalmology, Keio University School of Medicine, Tokyo, Japan; 2 Department of Ophthalmology, Juntendo University Graduate School of Medicine, Tokyo, Japan; 3 Department of Ophthalmology, Jikei University School of Medicine, Tokyo, Japan; 4 Department of Ophthalmology & Visual Sciences, Kyoto University, Kyoto, Japan; 5 Division of Medical Information Technology & Administration Planning, Kyoto University Hospital, Kyoto, Japan; 6 Department of Ophthalmology, Juntendo Tokyo Koto Geriatric Medical Center, Tokyo, Japan; 7 National Institute of Sensory Organs, National Hospital Organization Tokyo Medical Center, Tokyo, Japan; 8 Department of Ophthalmology, Kyorin University, Mitaka, Japan

## Abstract

To evaluate the association between vision-related quality of life (QoL), physical inactivity, and locomotor dysfunction in subjects with visual impairment.This cross-sectional study included 215 visually impaired subjects recruited from six ophthalmology departments in Japan. The physical inactivity and locomotor dysfunction associated with their visual impairment was investigated. The physical activity level was assessed using the short form of the International Physical Activity Questionnaire and classified as high, moderate, or low. Locomotor function was evaluated with the Geriatric Locomotive Function Scale. Vision-related QoL was evaluated using the 25-item National Eye Institute Visual Function Questionnaire. Background data, including for age, sex, best-corrected visual acuity for each eye, causative eye diseases, systemic comorbidities, and body mass index, were also collected.The average patient age was 69.6 (range, 20–93 years; standard deviation, 14.5 years) and 118 patients (54.9%) were men. Multivariate analysis showed that vision-related QoL and best-corrected visual acuity in the worse eye were significantly associated with physical inactivity and that vision-related QoL, female sex, age, and presence of systemic comorbidity were significantly associated with locomotor dysfunction. Vision-related quality of life is associated with physical inactivity and locomotor dysfunction.

## Introduction

Visual impairment is an increasingly prevalent public health problem, particularly in aging populations. An estimated 1.64 million individuals in Japan and 3.22 million in the US have impaired vision^1,2^. This number is expected to increase in developed countries because of the increased incidence of age-related eye diseases, such as glaucoma, macular degeneration, and diabetic retinopathy^1–4^.

Vision is a key element of health and quality of life (QoL)^5,6^. Blindness and visual impairment are among the 10 most common causes of disability in the US and are associated with both diminished QoL and a shorter life expectancy^7,8^. Individuals with impaired vision have an increased risk of road traffic accidents and are more likely to cease or curtail driving^9,10^. Visual impairment has also been associated with emotional distress^11,12^ and infrequent socialization^13,14^. Thus, visually impaired individuals may have functional and psychosocial burdens that affect various aspects of their daily lives. Further, visually impaired older adults have difficulties with physical functioning^15,16^, which increases the risk of falls and fractures^17–23^. A fear of falls and perceived risks of mobility have been reported to limit physical activity in visually impaired patients^24–26^.

Physical inactivity has been identified as a critical health problem during the last decade^27^. Restrictions in physical activity are associated with decreased QoL and higher rates of morbidity and mortality^28,29^. The Japanese Orthopedic Association has advanced the concept of “locomotive syndrome”, ie, a condition of reduced mobility resulting from impairment of the organs involved in locomotion^30,31^. In an aging society, such as that in Japan, locomotor dysfunction has a major impact on public health.

Compared with their sighted counterparts, blind children and adolescents have more sedentary lifestyles and lower levels of physical fitness^32,33^. Similar relationships have been found in older adults. Visually impaired older individuals tend to choose less active lifestyles because of difficulty with social interaction^34^. A few studies have investigated physical activity levels and locomotor function in relation to degree of visual impairment^35,36^. However, there are no reports on the association between vision-related QoL and physical activity/locomotor function in adults with visual impairment. The aims of this study were to assess physical activity and locomotor function in visually impaired patients and to determine their relationship with vision-related QoL.

## Patients and Methods

### Design, setting, and patients

This study was part of a nested research project entitled “Health Economics Research on Vision Screening for Adults” funded by the Ministry of Health, Labour and Welfare of Japan. The study data were drawn from a multicenter survey conducted between September 2011 and December 2012 as part of this larger project. Two hundred and fifteen Japanese patients with visual impairment were recruited from the ophthalmology departments of the National Hospital Organization Tokyo Medical Center, Keio University School of Medicine, Juntendo University, Juntendo Tokyo Koto Geriatric Medical Center, Jikei University, and Kyoto University.

For evaluation of vision-related QoL, each patient underwent a clinical assessment and completed the Japanese version of the 25-item National Eye Institute Visual Function Questionnaire (VFQ-25), including optional questions. Physical activity and locomotor function were evaluated using the short form of the International Physical Activity Questionnaire (IPAQ-J) and the short version of the Geriatric Locomotive Function Scale (GLFS-5), respectively.

We included adult patients, aged 20 years or older, with ocular conditions that resulted in visual impairment and a best-corrected visual acuity (BCVA) of ≤0.6 in at least one eye. These inclusion criteria were set according to the classification of visual impairment in the Japanese Welfare of Physically Disabled Persons Act. We included patients with chronic and invariable conditions due to any ophthalmic disease, along with those who had chronic systemic diseases such as hypertension and diabetes mellitus. Patients with acute ophthalmic disease states were excluded, as were those with severe systemic disease, dementia, psychiatric illness, and severe functional impairment, such as paralysis or limb defects. Presence of systemic comorbidity was defined based on self-reporting by the study participants. Body mass index (BMI) was calculated as weight (kg) divided by height squared (m^2^). We recruited patients who met the inclusion/exclusion criteria asked to participate in a consecutive fashion.

The study was conducted in accordance with the tenets of the Declaration of Helsinki and the Ethical Guidelines for Medical and Health Research Involving Human Subjects in Japan. The research protocol was approved by the Institutional Review Board at each clinical site, ie, Keio University, Tokyo Medical Center, Juntendo University, Juntendo Tokyo Koto Geriatric Medical Center, Jikei University, and Kyoto University. Written informed consent was obtained from all participants.

### Ophthalmic findings and visual acuity

Ophthalmic findings were obtained from the patients’ physicians. The ophthalmic diseases causing visual impairment were categorized as corneal disease, lens disease, glaucoma, diabetic retinopathy, macular degeneration, degenerative myopia, retinitis pigmentosa, amblyopia, optic nerve atrophy, and other.

Best corrected visual acuity (BCVA) was determined with the best optical lens equipped at each facility. BCVA was examined at a distance of 5 m, and values <0.01 were expressed as follows: counting fingers, categorized as an acuity of 0.004, hand motion as 0.002, light perception as 0.001, and no light perception as 0.0005^37^. Decimal visual acuity values were converted to logarithm of the minimum angle of resolution (logMAR) units for statistical analysis. For each patient, the BCVA for both eyes was subdivided into that for the better eye and that for the worse eye.

### Quality of life (VFQ-25)

The VFQ-25 was developed to assess QoL in visually impaired individuals^38^ and has been validated in many countries, including Japan^39^. It is used as a standard questionnaire for a wide range of patient populations, including the elderly and visually impaired. The VFQ-25 consists of 25 questions and 13 optional items organized into 12 subscales (one assessing general health and the remainder targeting vision-specific functions) with scores ranging from 0 to 100. Lower scores indicate lower QoL. The composite score, i.e., the average score across the 11 subscales related to vision-specific functions, was used in this study as a summary of vision-related QoL.

### Physical activity (IPAQ-J)

The short form of the IPAQ-J was used to determine physical activity during leisure time, domestic work, paid or unpaid work, and transport-related activities^40,41^. Patients were questioned about whether they engaged in walking, moderate-intensity activity, or vigorous-intensity activity at any time during their daily routine. Total scores for each type of activity were calculated by summing the scores for duration and frequency. The volume of activity was then calculated by weighting each type of activity by its energy requirements. This volume was defined in metabolic equivalent units (METs), and a weekly MET score (in minutes/week) was calculated. Total METs (in minutes/week) were calculated by summing all the scores for each type of activity. The level of physical activity (high, moderate, or low) was determined based on the scores calculated for both total volume and number of sessions in the IPAQ analysis algorithms^40,41^.

### Locomotor function (GLFS-5)

The GLFS-25 was designed to detect individuals with locomotive syndrome, a high-risk condition of locomotor dysfunction^42^. The GLFS-5 is the short version of the GLFS-25 and consists of five questions. The reliability and validity of the GLFS-25 and GLFS-5 have been verified in elderly Japanese individuals^31,42,43^. Several studies have shown that this questionnaire is valid for early detection of locomotive syndrome. GLFS-5 scores range from 0 to 20, with a higher score indicating a higher risk of locomotor dysfunction.

### Statistical analysis

The data were summarized as numbers with percentages for categorical variables and as means with the standard deviations or medians and the interquartile ranges (IQRs) for continuous variables. We used univariate and multivariate ordinal logistic regression analysis to assess the association between physical activity and each variable (sex, age, VFQ-25 score, BCVA in the better eye, BCVA in the worse eye, presence of systemic comorbidity, and BMI), because the level of physical activity was rated using a graded scale (high/moderate/low). With the ordinal logistic regression analysis, the equal slope assumption of the effect of variables with a significant ordinal trend was also assessed. *A p*-value ≥ 0.05 suggests that the equal slopes assumption holds, meaning that the effect of the independent variables is equal between scores adjacent to each other and indicating a meaningful ordinal logistic regression analysis. Univariate and multivariate logistic regression analysis was used to evaluate the association between the presence of locomotor dysfunction and each variable. The presence of locomotor dysfunction was confirmed by a score of ≥6 on the GLFS-5. The data were analyzed using Stata release 14 software (StataCorp LP; College Station, TX, USA). All *p*-values reported are two-tailed. A *p-*value < 0.05 was set as the threshold for statistical significance.

## Results

The patient demographic data are summarized in Table 1. The average age was 69.6 ± 14.5 (range, 20–93) years and 118 patients (54.9%) were men. The average BCVA in the better eye was 0.47 ± 0.63 logMAR and that in the worse eye was 1.55 ± 1.04 logMAR. The main diseases causing visual impairment were macular degeneration (n = 49; 22.8%), diabetic retinopathy (n = 33; 15.4%), glaucoma (n = 30; 14.0%), corneal diseases (n = 17; 7.9%), retinitis pigmentosa (n = 14; 6.5%), and degenerative myopia (n = 10; 4.7%). The other category (n = 45; 20.9%) included retinal vein occlusion, uveitis, chorioretinal atrophy, macular dystrophy, and phthisis with unknown etiology. One hundred and forty-seven patients (68.7%) had systemic comorbidity.

**Table 1:** Patient demographics and ophthalmic characteristics (n = 215).

	Mean (SD) or %	Range or cases (%)
Male sex (n, %)	118 (54.9)	
Age (years)	69.6 (14.5)	20–93
BCVA in better eye (logMAR)		NLP -1.5
	0.47 (0.63)	−0.18, −3.30
BCVA in worse eye (logMAR)	1.55 (1.04)	NLP -1.2 −0.08, −3.30
Diagnosis	AMD	49 (22.8)
	Diabetic retinopathy	33 (15.4)
	Glaucoma	30 (14.0)
	Corneal disease	17 (7.9)
	Retinitis pigmentosa	14 (6.5)
	Degenerative myopia	10 (4.7)
	Amblyopia	6 (2.8)
	Lens disease	5 (2.3)
	Optic disc atrophy	6 (2.8)
	Others	45 (20.9)
Systemic comorbidity		147 (68.7)
Body mass index	23.05 (3.65)	15.62–35.56

Abbreviations: AMD, age-related macular degeneration; BCVA, best-corrected visual acuity; logMAR, logarithm of the minimum angle of resolution; NLP, no light perception; SD, standard deviation.

A summary of the VFQ-25, IPAQ, and GLFS-5 scores is provided in Table 2. The mean VFQ-25 score was 54.4 ± 21.6. The average IPAQ score was 2212 ± 3782.6 METs/week. On the basis of IPAQ grade, physical activity levels were classified as low in 88 (40.9%) of the 215 subjects, moderate in 85 (39.5%), and high in 42 (19.5%). The mean GLFS-5 score was 6.3 ± 5.5, and 93 subjects (43.3%) were classified as at risk of locomotor dysfunction.

**Table 2:** Summary of questionnaire data.

	Mean (SD)	Median (IQR)	Observed range	Possible range
VFQ-25* (composite 11)	54.42 (21.55)	55.2 (40.0–72.3)	5.5–95.2	0–100
IPAQ (METs)	2211.7 (3782.6)	924.0 (198.0–2445.0)	0–28104.0	0–∞
GLFS-5**	6.29 (5.45)	4 (2–10)	0–20	0–20
		n	%	
IPAQ grade	Low	88	40.9	
	Moderate	85	39.5	
	High	42	19.5	
GLFS-5 score	≥6	93	43.3	
	<6	122	56.7	

Notes: *Higher values indicate better health. **Lower values indicate better health. A score ≥ 6 indicates suspected locomotive syndrome. Abbreviations: GLFS-5, Geriatric Locomotive Function Scale; IPAQ, International Physical Activity Questionnaire; IQR, interquartile range; MET, metabolic equivalent; SD, standard deviation; VFQ-25, 25-item National Eye Institute Visual Function Questionnaire.

We analyzed the factors associated with physical activity determined by the IPAQ using univariate and multivariate ordinal logistic regression models (Table 3**)**. In univariate analysis, there was a significant association between the physical activity (IPAQ) score and the VFQ-25 score, BCVA in the better eye, and BCVA in the worse eye. However, subsequent multivariate ordinal logistic regression analysis identified a significant association of physical activity with VFQ-25 score (adjusted odds ratio [aOR] 1.05; 95% confidence interval [CI] 1.03–1.07; *p* < 0.001) and BCVA in the worse eye (aOR 0.68; 95% CI 0.50–0.92; *p* = 0.01). The approximate likelihood-ratio test of proportionality of odds across categories was not statistically significant (*p* = 0.30), indicating that there was no difference in the coefficient among the pairs, confirming that this method of analysis was appropriate for this study.

**Table 3:** Odds ratios and confidence intervals for demographic variables according to physical activity (IPAQ) using ordinal logistic regression analysis (n = 215).

Variable	Univariate analysis				Multivariate analysis			
	OR	95% CI		*p*-value	Adjusted OR	95% CI		*p*-value
Sex (male/female)	1.28	0.77	2.12	0.34	0.85	0.48	1.50	0.56
Age (per + 1 year old)	0.99	0.97	1.00	0.10	0.99	0.97	1.01	0.38
VFQ-25 composite 11 (per + 1 score)	1.05	1.03	1.06	<0.001	1.05	1.03	1.07	<0.001
BCVA in better eye (per + 1 logMAR VA)	0.31	0.19	0.52	<0.001	1.04	0.55	1.96	0.91
BCVA in worse eye (per + 1 logMAR VA)	0.60	0.46	0.78	<0.001	0.68	0.50	0.92	0.01
Systemic comorbidity (present vs absent)	0.59	0.35	1.01	0.05	0.92	0.49	1.75	0.80
BMI (per + 1 unit)	0.94	0.88	1.01	0.09	1.00	0.92	1.08	0.94

Abbreviations: BCVA, best-corrected visual acuity; BMI, body mass index; CI, confidence interval; IPAQ, International Physical Activity Questionnaire; logMAR, logarithm of the minimum angle of resolution; OR, odds ratio; VA, visual acuity; VFQ-25, 25-item National Eye Institute Visual Function Questionnaire.

The factors associated with locomotor dysfunction determined by the GLFS-5 were analyzed using univariate and multivariate logistic regression models (Table 4). There was a significant association between the GLFS-5 score and age, VFQ-25 score, BCVA in the better eye, BCVA in the worse eye, systemic comorbidity, and BMI in the univariate analysis. However, subsequent multiple logistic regression analysis identified significant associations of locomotor dysfunction with female sex (aOR 2.64; 95% CI 1.11–6.27; *p* < 0.03), age (aOR 1.09; 95% CI 1.04–1.13; *p* < 0.001), VFQ-25 score (aOR 0.92; 95% CI 0.90–0.95; *p* < 0.001), and presence of systemic comorbidity (aOR 6.63; 95% CI 2.44–17.98; *p* < 0.001).

**Table 4:** Odds ratios and confidence intervals for demographic variables according to locomotor function (GLFS-5) using logistic regression analysis (n = 215).

Variable	Univariate analysis				Multivariate analysis			
	OR	95% CI		*p*-value	Adjusted OR	95% CI		*p*-value
Sex (female/male)	1.08	0.63	1.86	0.77	2.64	1.11	6.27	0.03
Age (per + 1 year old)	1.07	1.04	1.11	<0.001	1.09	1.04	1.13	<0.001
VFQ-25 composite11 (per + 1 score)	0.94	0.92	0.96	<0.001	0.92	0.90	0.95	<0.001
BCVA in better eye (per + 1 logMAR VA)	3.36	1.94	5.82	<0.001	0.62	0.27	1.42	0.25
BCVA in worse eye (per + 1 logMAR VA)	1.49	1.14	1.95	<0.01	1.33	0.87	2.04	0.20
Systemic comorbidity (present vs absent)	7.19	3.41	15.17	<0.001	6.63	2.44	17.98	<0.001
BMI (per + 1 unit)	1.09	1.00	1.17	0.04	1.06	0.95	1.19	0.31

Abbreviations: BCVA, best-corrected visual acuity; BMI, body mass index; CI, confidence interval; GLFS-5, Geriatric Locomotive Function Scale; MAR, logarithm of the minimum angle of resolution; OR, odds ratio; VA, visual acuity; VFQ-25, 25-item National Eye Institute Visual Function Questionnaire.

## Discussion

In this cross-sectional study, we assessed physical activity levels and locomotor dysfunction in visually impaired individuals. The IPAQ was used to evaluate physical activity and the GLFS-5 to estimate the risk of locomotor dysfunction. We found a significant association of vision-related QoL determined by the VFQ-25 score and BCVA in the worse eye with physical activity (IPAQ) in multivariate analysis. Locomotor dysfunction evaluated by the GLFS-5 had a significant association with vision-related QoL, as well as female sex, age, and systemic comorbidity.

Low levels of physical activity are widely recognized to be associated with undesirable health consequences, including higher rates of morbidity and mortality^27–29^. Moreover, physical inactivity in individuals with low vision has recently attracted attention^34–36^. Our results are consistent with recent reports showing an association between visual impairment and low levels of physical activity/mobility. We found that BCVA in the better eye was not associated with the physical activity level or locomotor function. This result was unexpected because vision-related QoL is closely related to BCVA in the better eye in general^5,44–46^. Instead, we found that BCVA in the worse eye was significantly associated with the physical activity level. Vision consists of multi-dimensional aspects, including BCVA in both eyes, visual fields, binocular vision, color discrimination, and contrast sensitivity. It is reported that various types of vision problems, such as decreased vision in the worse eye, incomplete stereopsis, and decreased contrast sensitivity, have a significant impact on the risk of hip fracture^47^. Therefore, vision-related QoL may be superior to BCVA in the better eye alone to estimate the effects of vision on physical activity. Another possible explanation is that all subjects in our study cohort were visually impaired, which may have limited our ability to find an association.

Female sex, age, and systemic comorbidity were associated with an increased risk of locomotor dysfunction in addition to vision-related QoL. The prevalence of osteoporosis at the lumbar spine and femoral neck in women was reported to be six-fold and two-fold higher, respectively, than in men (*p* < 0.001)^48^. Further, in Japan, the prevalence of osteoarthritis of the knee is significantly higher in women than in men (61.5% vs 42.0%; p < 0.001). Our findings also suggest a higher prevalence of locomotor dysfunction in women. It seems reasonable that systemic comorbidity has a significant impact on locomotor dysfunction. Grue *et al*.^49^ reported that a combination of vision and hearing impairments increased the risk of hip fracture.

The number of visually impaired individuals is increasing in the aging populations of developed countries^1,2^. Moreover, physical inactivity and locomotor dysfunction can lead to elderly individuals becoming bedridden^27^. Visually impaired individuals have lower vision-related QoL, which in turn may lead to lower levels of physical activity^35,36^. The level of visual perception can influence participation in physical activity, and the fear of falls and perceived risks of mobility may further limit physical activity^24–26^. Long-term physical inactivity may eventually worsen general health, which can lead to physical disability^50^. Further, worsening of systemic conditions, such as diabetes and dialysis, can exacerbate vision problems. Thus, vision problems, physical inactivity, and physical disability may form a vicious cycle.

The results of our study suggest that visual function is critical for not only preservation of vision-related QoL but also maintenance of physical health. Maintaining normal sight in aging adults should be emphasized in vision care programs, and physical activity should be encouraged in elderly patients with vision problems to maintain or improve their general health^31,51^. At the same time, safe strategies to improve mobility and increase physical activity in visually impaired individuals should be developed^36^. The findings of this study show an important association between physical activity, locomotor function, and vision-related QoL. Therefore, interventions that improve vision-related QoL may be able to activate physical activity and at the same time lead to improvement of locomotor function in adults. Further, greater participation in physical activity should be encouraged and facilitated in visually impaired adults.

Our study had some limitations. First, we only investigated visually impaired subjects recruited from six ophthalmology departments in Japan, and the ophthalmic diseases causing visual impairment in this cohort might not be a representative sampling of causative diseases in the Japanese population. In addition, there is no data of the subjects without visual impairment. We should compare data between subjects between with and without visual impairment in further study. Second, we used self-reported questionnaires to assess physical activity and locomotive syndrome, and recall bias could have affected our results and influenced our conclusions. We suggest that future studies use objective techniques, such as accelerometry, to evaluate physical activity and objective evaluations to characterize physical disability^36^. Information on lifestyle is also needed, such as whether patients live alone or with family. Another limitation of this study is its cross-sectional design, which could not establish a causal relationship between vision-related QoL and physical inactivity/locomotor dysfunction. It is likely that these subjects could have been less physically active in their earlier years leading to systemic disorders such as cardiovascular disease and diabetes that have shared pathogenesis with visual impairments. So in turn physical inactivity in younger years may result in visual impairment and not necessarily the other way around. Furthermore, in this study we excluded subjects with severe systemic diseases. The effect of vision-related QOL on the IPAQ scores of such patients is considered to be mild. We should proceed with more research to elucidate these points.

## Conclusion

In summary, our present findings show that visually impaired patients have low levels of physical activity and are at high risk for locomotor dysfunction. We believe that the results of this study raise a novel viewpoint that supplements interventional approaches to visual impairment. Further research on physical activity and locomotor dysfunction in visually impaired individuals is required to elucidate the circular relationship suggested by our results, and greater participation in physical activity should be encouraged and facilitated in visually impaired individuals.
